# 30. AN IMMUNE PATHOGENESIS OF PSYCHOSIS? EVIDENCE AND CHALLENGES FROM BENCH TO BEDSIDE

**DOI:** 10.1093/schbul/sby014.121

**Published:** 2018-04-01

**Authors:** Rachel Upthegrove

**Affiliations:** University of Birmingham

## Abstract

**Overall Abstract:** The immune pathogenesis story of schizophrenia is gathering momentum, with increasing evidence from animal models, genetic, circulating biomarker and neuropathological studies. Potentially ground breaking new treatment approaches are proposed. However, it is vital that basic science and is equally matched by deep understanding of the complexity of clinical samples and management of multiple confounding factors when moving from bench to bedside. This presentation will pull together key speakers from a variety of fields, demonstrating the need for continued dialogue in translational, and reverse translational, approach. We will present findings from preclinical studies, genetic insights, longitudinal modelling of immune markers from population-based samples and detailed analysis from clinical samples. Data will include evidence of a prenatal immune activation and the potential transgenerational transmission of behavioural and neuronal abnormalities, co-variation of gene sets associated with both increased risk of schizophrenia and immune function (eg CSMD1, DPP4) together with CRP and peripheral inflammatory cytokine association with symptom profiles in both larger population and clinical samples. Thus, evidence presented will move from large data to fine grain analysis, animals to man and from bench to bedside.

We aim to provide insights into early pathophysiological processes and forward avenues of research to the ultimate aim of elucidating the immune dysfunction impact on psychosis and future avenues for effectiveness of treatment.

